# Clinical efficacy of spleen-preserving distal pancreatectomy with or without splenic vessel preservation: A Meta-analysis

**DOI:** 10.1097/MD.0000000000008600

**Published:** 2017-12-01

**Authors:** Ningning Sun, Guangjun Lu, Likun Zhang, Xiaoling Wang, Chunmin Gao, Jieliang Bi, Xiaoyi Wang

**Affiliations:** Surgical Department, Wei Fang Traditional Chinese Hospital, Weifang, China.

**Keywords:** meta-analysis, splenic vessel preservation, splenic vessel resection

## Abstract

**Objective:**

The meta-analysis was performed to investigate the clinical efficacy of spleen-preserving distal pancreatectomy with splenic vessel preservation (SPDP-SVP) and spleen-preserving distal pancreatectomy with splenic vessel resection (SPDP-SVR).

**Methods:**

Potential articles were searched on the databases of Pubmed, Embase, and Chinese National Knowledge Infrastructure (CNKI) from January 1988 until March 2017. Weight mean difference (WMD) with 95% confidence interval (CI) was applied to compare the efficacy of SPDP-SVP and SPDP-SVR. Odds ratio (OR) with 95% CI was calculated to figure out the risks for complications. *P*< .05 or *I*^*2*^>50% indicated significant heterogeneity. The random-effects model is used to pool data if significant heterogeneity exists; otherwise, the fixed-effects model is used. Publication bias was evaluated by Begg's funnel plot.

**Results:**

Thirteen eligible articles were obtained in the meta-analysis. SPDP-SVP seemed to relate with reduced operative time and blood loss, prolonged hospital stay, and less complications; however, the effects were not statistically significant. Meanwhile, we found that SPDP-SVP was closely related with the reduced rate of splenic infarction and gastric varices (OR = 0.16, 95% CI = 0.09–0.29; OR = 0.08, 95% CI = 0.02–0.35). No publication bias was observed in the analysis (*P* = .636).

**Conclusions:**

SPDP-SVP seems to show superiority than SPDP-SVR in reducing the rate of splenic infarction and gastric varices.

## 1 Introduction

Distal pancreatectomy with splenectomy has been demonstrated to be an effective therapy for managing benign or low-grade malignant lesions in the body or tail of the pancreas.^[1]^ However, splenectomy is usually related with high risk of sepsis and poor survival.^[2]^ Hence, spleen-preserving distal pancreatectomy (SPDP) is frequently applied to decrease the occurrence of sepsis and improve patients’ survival.^[3]^

Two surgical techniques for SPDP have been described. Spleen-preserving distal pancreatectomy with splenic vessel preservation (SPDP-SVP) preserves the main splenic artery and vein and excises the tail of the pancreas and those small, short vascular connections to the body.^[4,5]^ For this technique, a small breakage in the splenic vessels could result in massive intraoperative bleeding, which makes SPDP difficult. To control bleeding from the main splenic vessels, combined spenectomy is always suggested. Another one is spleen-preserving distal pancreatectomy with splenic vessel resection (SPDP-SVR). It involves resection of the splenic vein and artery before distal pancreatectomy, and conservation of the short splenocolic and gastric vessels to keep normal blood flow of spleen. During the procedure, not only the 2 main splenic vessels need to be controlled, but also fine resection near the splenic hilum is always required. Moreover, the risks of perigastric varices and spleen-related morbidities should be bewared.^[6,7]^ Up to now, the superiority of SPDP-SVP or SPDP-SVR in managing the pancreatic lesions is still debatable.

The current meta-analysis was initiated to compare the clinical efficacy between SPDP-SVP and SPDP-SVR in the management of the pancreatic lesions. In the analysis, laparoscopic, robotic and open surgery SPDP were all considered. The outcome contributes to improving the treatment of patients with benign or low-grade malignant lesions in the body or tail of the pancreas.

## 2 Methods

### 2.1 Search strategy

We searched Pubmed, Embase, and Chinese National Knowledge Infrastructure (CNKI) databases for potential articles from January 1988 until March 2017. The search terms included distal pancreatectomy, spleen, vessel, preservation/conservation, and Kimura technique. The references of obtained articles were manually searched to identify possible studies.

### 2.2 Inclusion and exclusion criteria

These obtained articles were selected according to inclusion criteria and exclusion criteria. The studies comparing the clinical efficacy of SPDP-SVP and SPDP-SVR will be considered in the current meta-analysis, whatever approach (robotic-assisted, laparoscopic, or open) was used. For the studies with overlapped data, the recent published one was selected.

The exclusion criteria were as follows: review articles; case reports; the studies only focused on the clinical efficacy of SPDP-SVP or SPDP-SVR and not compared SPDP-SVP and SPDP-SVR; and the articles not reported available data.

### 2.3 Data extraction

Two authors were responsible for extracting data from included articles. The data were: name of first author, publication year, country, sample size, study design (retrospective, prospective, or randomized controlled trial), surgery type (robotic-assisted, laparoscopic, or open), operative time, blood loss, hospital time, overall complications (minor complications and major complications), pancreatic fistula, splenic infarction, and gastric varices of each group. Minor complication is graded as Dindo grades I-II and major complication is listed as Dindo grades III-IV. The debatable issues were discussed with a third author.

### 2.4 Statistical analysis

The current meta-analysis was performed with Stata 12.0 software. Weight mean difference (WMD) for continuous outcomes and odds ratios (ORs) for dichotomous outcome are provided. WMD with 95% confidence interval (CI) was used to figure out the influences of SPDP-SVP in operative time, blood loss, and hospital stay, compared with SPDP-SVR. OR with 95% CI was calculated to represent the risks for overall complications. *P*< .05 or *I*^*2*^> 50% indicated significant heterogeneity. The random-effects model is used to pool data if significant heterogeneity exists; otherwise, the fixed-effects model is used. Sensitivity analysis was performed to evaluate the robustness of overall results. Possible publication bias was evaluated by Begg's funnel plot and Egger's regression analysis.

## 3 Results

### 3.1 Selection of eligible articles

**Figure 1:**
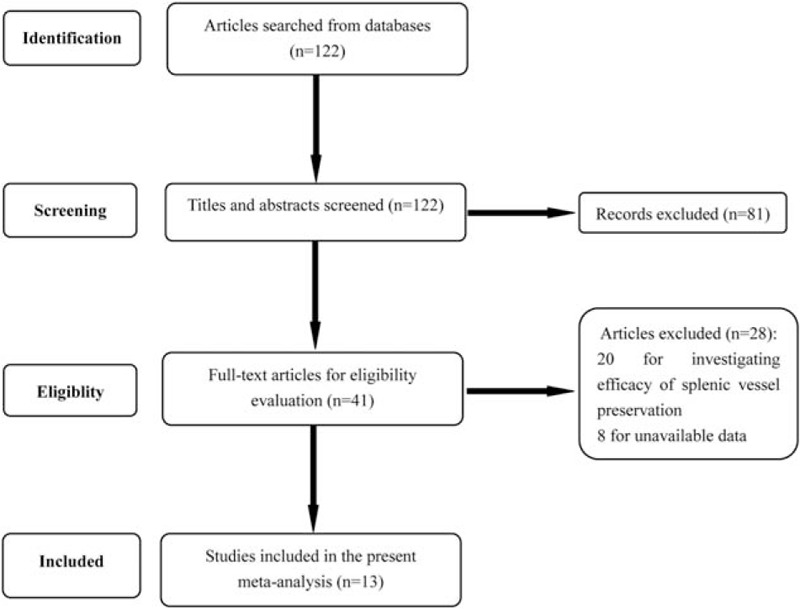
Articles selection process. 13 eligible articles were included in the present meta-analysis.

We carefully selected the obtained articles according to the inclusion and exclusion criteria. At last, 13 eligible articles were obtained.^[8–18,28,29]^ About 122 relevant articles were obtained after rough search. And 81 articles were excluded after screening titles and abstract. For the remaining 41 articles, 31 articles were removed for not comparing the efficacy of SPDP-SVP and SPDP-SVR (n = 20) and unavailable data (n = 8). The selection process was exhibited in Figure 1. The detailed information of included articles was listed in Table 1.^[8–18,28,29]^

**Table 1:**
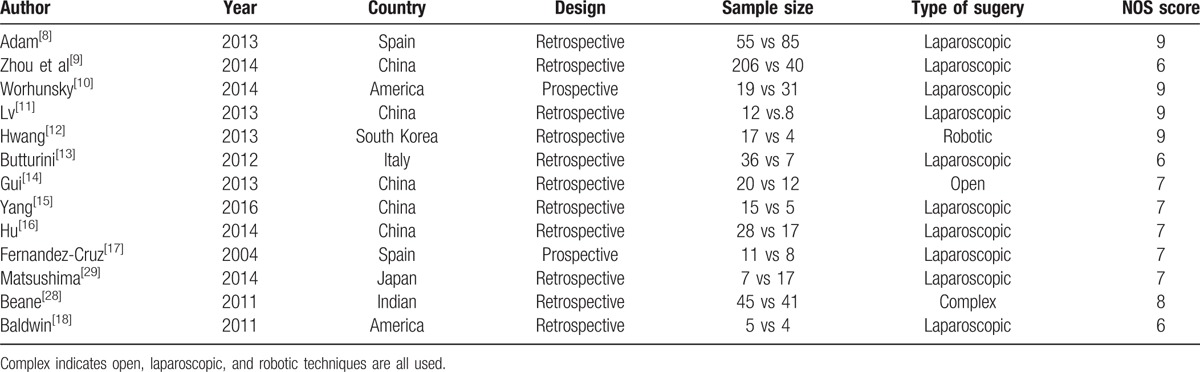
Basic information of included studies.^[8–18,28,29]^.

### 3.2 Comparison in operative time, blood loss, hospital stay, and complications (overall complications, pancreatic fistula, splenic infarction,and gastric varices) between SPDP-SVP and SPDP-SVR

**Figure 2:**
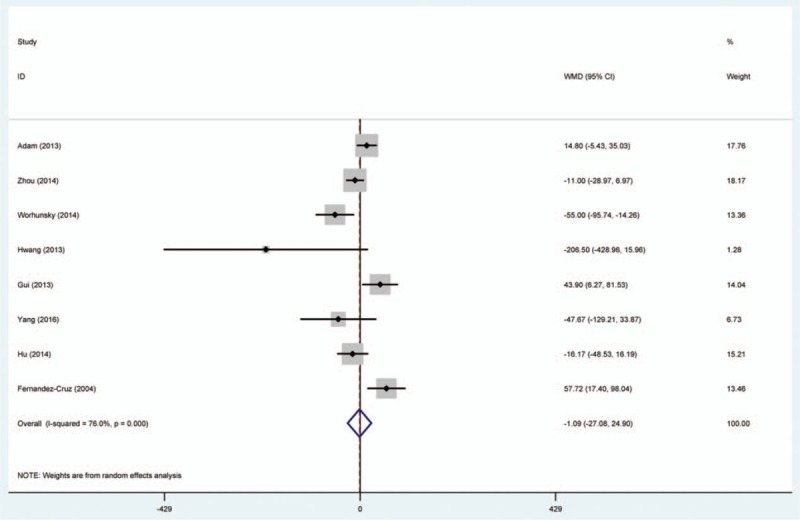
Comparison between SPDP-SVP and SPDP-SVR in operative time.

**Figure 3:**
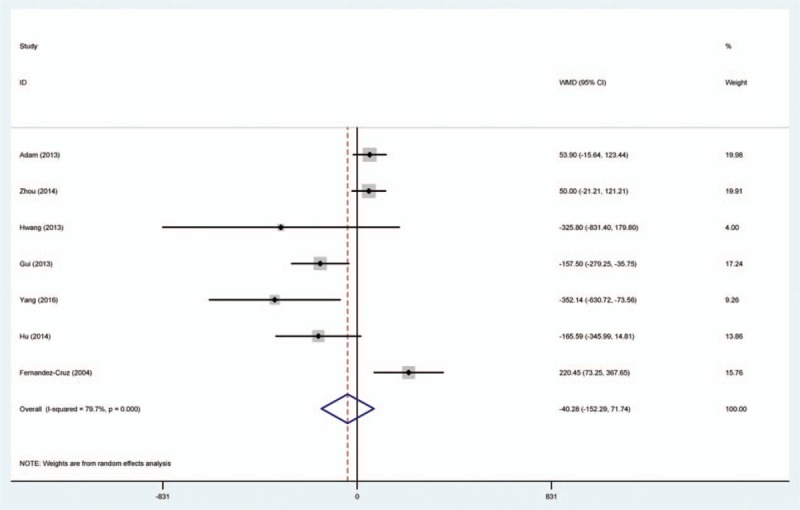
Comparison between SPDP-SVP and SPDP-SVR in blood loss.

**Figure 4:**
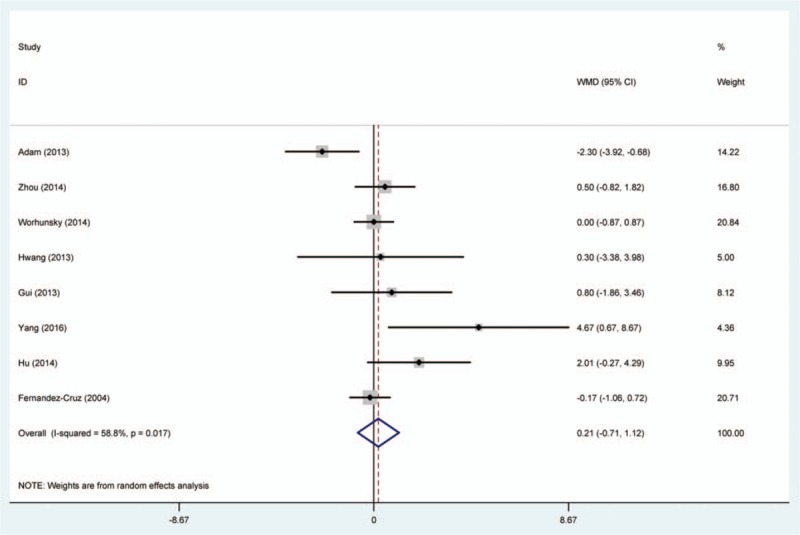
Comparison between SPDP-SVP and SPDP-SVR in hospital stay.

Random-effects model was used to comparing the efficacy of SPDP-SVP and SPDP-SVR in operative time, blood loss, and hospital stay (Table 2, Figs. 2–4). We found that SPDP-SVP seemed to reduce the operative time and blood loss, compared to SPDP-SVR (WMD: –1.09 and –40.28); however, the effects were not statistically significant. Meanwhile, SPDP-SVP seemed to relate with prolonged hospital stay (WMD: 0.21, 95% CI: –0.71, 1.12), but the relationship was not statistically significant.

**Table 2:**
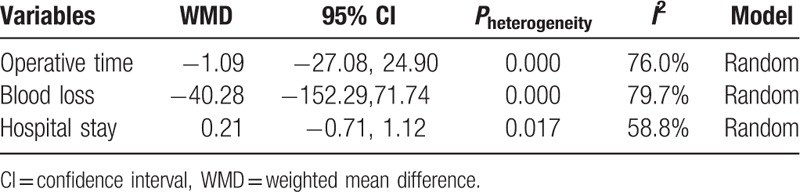
Pooled results of operative time, blood loss, and hospital stay.

**Figure 5:**
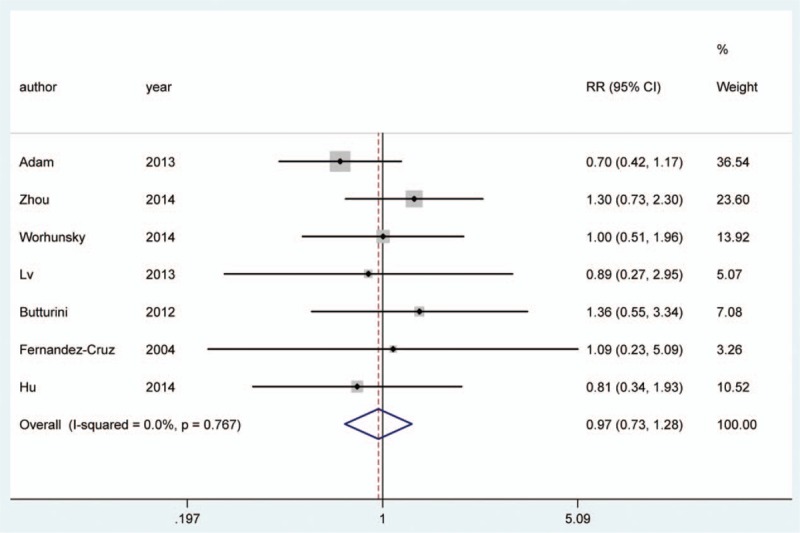
Comparison between SPDP-SVP and SPDP-SVR in complications.

**Figure 6:**
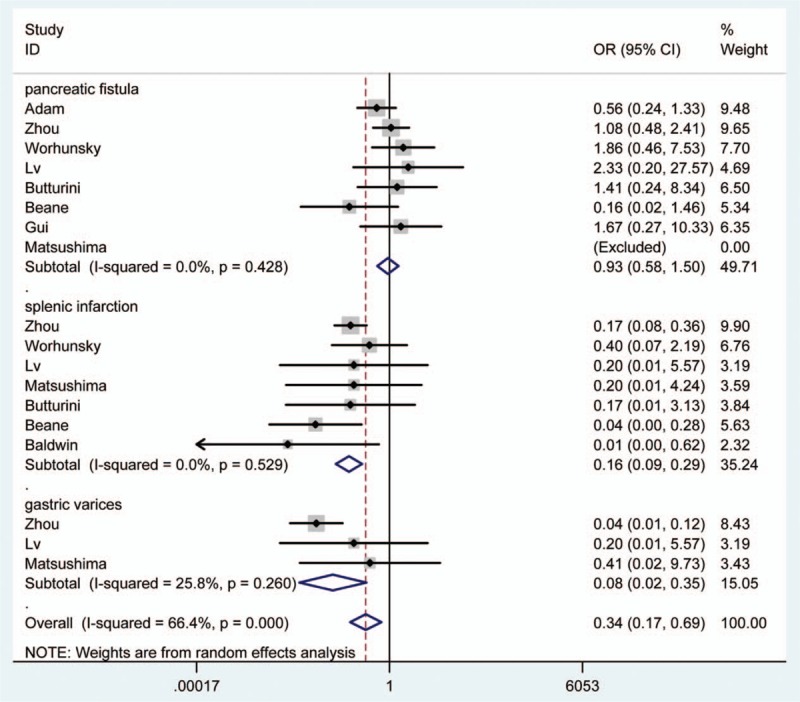
Comparison between SPDP-SVP and SPDP-SVR in pancreatic fistula, splenic infarction and gastric varices.

In addition, the outcome indicated that SPDP-SVP was related with less overall complications (OR: 0.97, 95% CI: 0.73, 1.28) and the relationship was not statistically significant (Table 3, Fig. 5). Meanwhile, we found that SPDP-SVP was closely related with the reduced rate of splenic infarction and gastric varices (OR = 0.16, 95% CI = 0.09–0.29; OR = 0.08, 95% CI = 0.02–0.35) (Fig. 6).

**Table 3:**
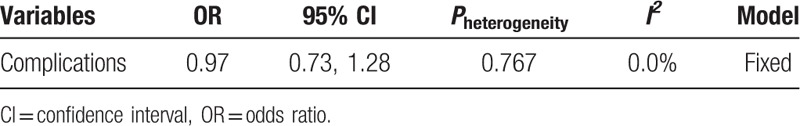
Pooled result of complications.

### 3.3 Sensitivity analysis

Sensitivity analysis was performed to assess the robustness of pooled outcome. The analysis suggested the pooled outcome was robust.

### 3.4 Publication bias detection

**Figure 7:**
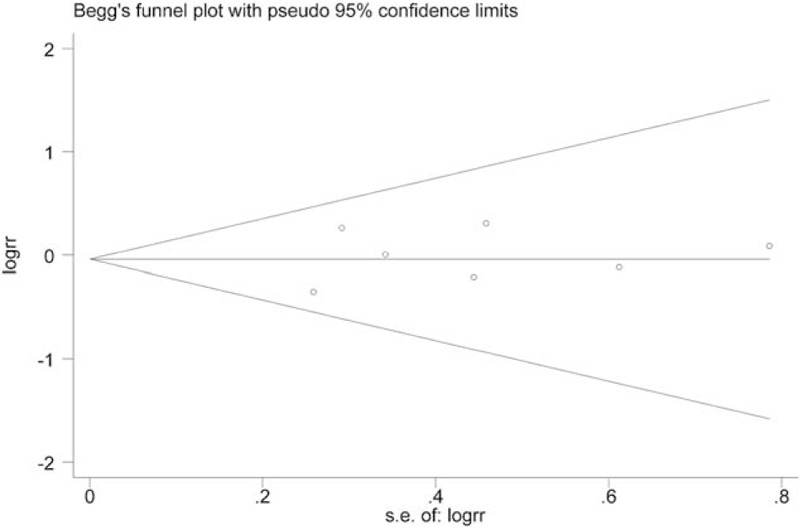
Begg's funnel plot.

Begg's funnel plot and Egger's regression analysis were adopted to evaluate the possible publication bias. No publication bias was observed in the current meta-analysis (*P* = .636, complications analysis) (Fig. 7).

## 4 Discussion

Distal pancreatectomy for malignant tumors of the tail or body of the pancreas needs a splenectomy. Whereas, splenectomy usually results in postsplenectomy infection in 1% to 5% patients and cause 50% of mortality rate.^[19]^ Besides, it may increase the postoperative platelet count.^[20]^ In 1943, Mallet-Guy and Vachon firstly described SPDP technique.^[21]^ It is commonly applied in patients with benign or low-grade malignant tumors in the body and tail of the pancreas. In 1994, Soper et al^[22]^ developed an animal model for laparoscopic distal pancreatectomy, which is demonstrated to be safe and effective. In 1996, Gagner et al^[23]^ suggested a spleen-preserving laparoscopic distal pancreatectomy procedure with preserving splenic artery and vein, which was performed in patients with cystic tumors, neuroendocrine tumors, and chronic pancreatitis. In 1999, Vezakis et al^[24]^ demonstrated that spleen-preserving laparoscopic distal pancreatectomy could be performed with splenic vessel resection as well.

As for SPDP-SVP, it could conserve the splenic artery and vein, maintain the blood supply to spleen, and ultimately reduce the risks of abscess formation and splenic necrosis. However, SPDP-SVP is time-consuming. Moreover, the technique is difficult because of delicately dissecting the small branches of splenic vessels. During SPDP-SVR procedure, dissecting the splenic vessels may be difficult when large tumors compress and distort the course of the vessels. SPDP-SVR has been demonstrated to be faster and less technically demanding compared with SPDP-SVP.^[25–27]^

In recent years, more attention has been paid to compare the clinical efficacy of SPDP-SVP and SPDP-SVR in managing the pancreatic lesions. Adam et al^[8]^ thought that the short-term benefits of SPDP-SVP compared with SPDP-SVR may lead to an increased preference for this technique. In the study of Zhou et al,^[9]^ there were no significant differences between SPDP-SVP and SPDP-SVR groups in blood loss and operative time. However, the rates of splenic infarction were 16.0% in the SPDP-SVP group and 52.5% in the SPDP-SVR group at 3 days after surgery. At 6 months, the rates of gastric varices were 1.9% in the SPDP-SVP group and 35% in the SPDP-SVR group. These data also indicated the superiority of SPDP-SVP than SPDP-SVR. Beane et al^[28]^ reported that SPDP-SVP procedure was related with less blood loss than SPDP-SVR. In addition, SPDP-SVP resulted in fewer grade B or C pancreatic fistulas and splenic infarctions, and shorter post-operative length of stay. They concluded that SPDP-SVP was preferred when SPDP was performed. On the contrary, the study by Matsushima et al^[29]^ suggested that SPDP-SVR (Warshaw) could be used as the more appropriate procedure in cases whose tumors are relatively large or close to the splenic vessels.

In the current meta-analysis, we found that SPDP-SVP seemed to reduce operative time and blood loss compared to SPDP-SVR; however, the effects were not statistically significant. Besides, SPDP-SVP was related with prolonged hospital stay (WMD: 0.21), but the relationship was not statistically obvious. Meanwhile, we found that SVP was related with less complications (RR: 0.97, 95% CI: 0.73, 1.28) and the relationship was not statistically significant. Moreover, we found that SPDP-SVP was closely related with the reduced rate of splenic infarction and gastric varices (OR = 0.16, 95% CI = 0.09–0.29; OR = 0.08, 95% CI = 0.02–0.35). The results were calculated based on 13 articles and they were credible. One meta-analysis by Tang et al^[30]^ reported that operative time, blood loss, postoperative complications, pancreatic fistula rates, and hospital stays were comparable between SPDP-SVP and SPDP-SVR. However, SPDP-SVR was related with higher incidence rates of splenic ischemia and gastric/perigastric varices. Another meta-analysis by Partelli et al^[31]^ concluded that the 2 procedures were comparable in terms of intraoperative blood loss and rate of pancreatic fistula. SPSP-SVR did not affect the risk of perigastric collateral vessels and submucosal varices. Compared with the 2 meta-analyses, the selected articles were searched on CNKI database and much more Chinese population was analyzed, which may contribute to obtaining much more comprehensive conclusion. In the analysis, both of overall complications and every complication were investigated, which may help for obtaining much more clear conclusions.

However, there existed limitations in the analysis. First, there existed significant heterogeneity in the analysis of operative time, blood loss and hospital time, which might result from the differences in characteristics of patients and operation manipulation. Second, specific type of complications was not analyzed in the current analysis because of the lack of sufficient data. Third, most studies were conducted with retrospective design in addition of 2 studies (Worhunsky et al^[10]^ and Fernandez-Cruz et al^[17]^). Meanwhile, no selected studies were designed as randomized controlled trials (RCTs). The doctors must respect the intention of patients and informed consent must be signed by patients before surgery, which may be one reason for no RCTs.

In conclusion, SPDP-SVP seems to show superiority than SPDP-SVR for patients with benign or low-grade malignant tumors in the body and tail of the pancreas. It shows close relationship with reduced rate of splenic infarction and gastric varices.

## References

[1] Nathan H Cameron JL Goodwin CR Risk factors for pancreatic leak after distal pancreatectomy. Ann Surg 2009;250:277–81. 19638926 10.1097/SLA.0b013e3181ae34be

[2] Moffett SL Overwhelming postsplenectomy infection: managing patients at risk. JAAPA 2009;22:36–9. 45. 10.1097/01720610-200907000-00009 19697570

[3] Fernández-Cruz L Orduña D Cesar-Borges G Distal pancreatectomy: en-bloc splenectomy vs spleen-preserving pancreatectomy. HPB (Oxford) 2005;7:93–8. 18333170 10.1080/13651820510028972 PMC2023931

[4] Kimura W Inoue T Futakawa N Spleen-preserving distal pancreatectomy with conservation of the splenic artery and vein. Surgery 1996;120:885–90. 8909526 10.1016/s0039-6060(96)80099-7

[5] Kimura W Moriya T Ma J Spleen-preserving distal pancreatectomy with conservation of the splenic artery and vein. World J Gastroenterol 2007;13:1493–9. 17461439 10.3748/wjg.v13.i10.1493 PMC4146889

[6] Tien YW Liu KL Hu RH Risk of varices bleeding after spleen-preserving distal pancreatectomy with excision of splenic artery and vein. Ann Surg Oncol 2010;17:2193–8. 20309639 10.1245/s10434-010-0983-6

[7] Miura F Takada T Asano T Hemodynamic changes of splenogastric circulation after spleen-preserving pancreatectomy with excision of splenic artery and vein. Surgery 2005;138:518–22. 16213907 10.1016/j.surg.2005.04.020

[8] Jean-Philippe A Alexandre J Christophe L Laparoscopic spleen-preserving distal pancreatectomy: splenic vessel preservation compared with the Warshaw technique. JAMA Surg 2013;148:246–52. 23682365 10.1001/jamasurg.2013.768

[9] Zhou ZQ Kim SC Song KB Laparoscopic spleen-preserving distal pancreatectomy: comparative study of spleen preservation with splenic vessel resection and splenic vessel preservation. World J Surg 2014;38:2973–9. 24968894 10.1007/s00268-014-2671-3

[10] Worhunsky DJ Zak Y Dua MM Laparoscopic spleen-preserving distal pancreatectomy: the technique must suit the lesion. J Gastrointest Surg 2014;18:1445–51. 24939598 10.1007/s11605-014-2561-x

[11] Lv GY Wang GY Jiang C Laparoscopic spleen-preserving distal pancreatectomy with or without splenic vessel conservation: a retrospective study of 20 cases. Hepatogastroenterology 2013;60:1785–8. 24624457

[12] Hwang HK Kang CM Chung YE Robot-assisted spleen-preserving distal pancreatectomy: a single surgeon's experiences and proposal of clinical application. Surg Endosc 2013;27:774–81. 23052527 10.1007/s00464-012-2551-6

[13] Butturini G Inama M Malleo G Perioperative and long-term results of laparoscopic spleen-preserving distal pancreatectomy with or without splenic vessels conservation: a retrospective analysis. J Surg Oncol 2012;105:387–92. 22025322 10.1002/jso.22117

[14] Xin G Ying L Yong C Comparison analysis between Kimura and Warshaw methods in spleen-preserving distal pancreatectomy (in Chinese). Acta Universitatis Medicinalis Nanjing 2013;10:1458–60.

[15] Zhou Y Clinical curative effect research of laparoscopic spleen-preserving distal pancreatectomy (in Chinese). Shandong University 2016.

[16] Wendi H Laparoscopic spleen-preserving distal pancreatectomy with and without splenic vessel preservation: a comparative study (in Chinese). Zhejiang University 2014.

[17] Fernández-Cruz L Martínez I Gilabert R Laparoscopic distal pancreatectomy combined with preservation of the spleen for cystic neoplasms of the pancreas. J Gastrointest Surg 2004;8:493–501. 15120376 10.1016/j.gassur.2003.11.014

[18] Baldwin KM Katz SC Espat NJ Laparoscopic spleen-preserving distal pancreatectomy in elderly subjects: splenic vessel sacrifice may be associated with a higher rate of splenic infarction. HPB (Oxford) 2011;13:621–5. 21843262 10.1111/j.1477-2574.2011.00341.x PMC3183446

[19] Brigden ML Overwhelming postsplenectomy infection still a problem. West J Med 1992;157:440–3. 1306065 PMC1011306

[20] Weledji EP Benefits and risks of splenectomy. Int J Surg 2014;12:113–9. 24316283 10.1016/j.ijsu.2013.11.017

[21] Mallet-Guy P Vachon A Pancreatites chroniques gauches. Paris: Masson; 1943.

[22] Soper NJ Brunt LM Dunnegan DL Laparoscopic distal pancreatectomy in the porcine model. Surg Endosc 1994;8:57–60. 8153866 10.1007/BF02909495

[23] Gagner M Pomp A Herrera MF Early experience with laparoscopic resections of islet cell tumors. Surgery 1996;120:1051–4. 8957494 10.1016/s0039-6060(96)80054-7

[24] Vezakis A Davides D Larvin M Laparoscopic surgery combined with preservation of the spleen for distal pancreatic tumors. Surg Endosc 1999;13:26–9. 9869683 10.1007/s004649900891

[25] Warshaw AL Conservation of the spleen with distal pancreatectomy. Arch Surg 1988;123:550–3. 3358679 10.1001/archsurg.1988.01400290032004

[26] Warshaw AL Distal pancreatectomy with preservation of the spleen. J Hepatobiliary Pancreat Sci 2010;17:808–12. 19882099 10.1007/s00534-009-0226-z

[27] Carrère N Abid S Julio CH Spleen-preserving distal pancreatectomy with excision of splenic artery and vein: a case-matched comparison with conventional distal pancreatectomy with splenectomy. World J Surg 2007;31:375–82. 17171488 10.1007/s00268-006-0425-6

[28] Beane JD Pitt HA Nakeeb A Splenic preserving distal pancreatectomy: does vessel preservation matter? J Am Coll Surg 2011;212:651–7. 21463805 10.1016/j.jamcollsurg.2010.12.014

[29] Matsushima H Kuroki T Adachi T Laparoscopic spleen-preserving distal pancreatectomy with and without splenic vessel preservation: the role of the Warshaw procedure. Pancreatology 2014;14:530–5. 25306307 10.1016/j.pan.2014.09.007

[30] Tang Y Tang SH Hu SY The efficacy of spleen-preserving distal pancreatectomy with or without splenic vessel preservation: a meta-analysis. Int J Clin Exp Med 2015;8:17128–39. 26770307 PMC4694207

[31] Partelli S Cirocchi R Randolph J A systematic review and meta-analysis of spleen-preserving distal pancreatectomy with preservation or ligation of the splenic artery and vein. The Surgeon 2016;14:109–18. 26723134 10.1016/j.surge.2015.11.002

